# Remote Learning in School Bands During the COVID-19 Shutdown

**DOI:** 10.1177/0022429420967008

**Published:** 2020-12-07

**Authors:** Phillip M. Hash

**Affiliations:** 1 Illinois State University, Normal, IL, USA

**Keywords:** eLearning, remote learning, distance learning, COVID-19, band

## Abstract

The global pandemic caused by the novel Coronavirus (COVID-19) in spring 2020 resulted in schools moving to remote learning (RL) models for the remainder of the academic year. The purpose of this study was to examine the practices, experiences, and perspectives of elementary and secondary school band directors in relation to RL during this period. Directors (*N* = 462) responded to survey questions related to several aspects of RL, including (a) technologies and materials, (b) activities and assessments, (c) student participation, (d) the challenges of teaching remotely, and (e) the extent to which experiences varied among participants in low-poverty versus high-poverty schools and at the elementary/middle school level versus high school level. I also examined (f) the conditions and practices of programs that experienced both high and consistent levels of student participation. Data indicated that the COVID-19 shutdown created many challenges for directors, particularly in schools with higher poverty levels and/or in rural locations. However, RL also created opportunities for instrumental teachers to incorporate into curricula (a) a wider range of technology; (b) more of a focus on individual musicianship; (c) lessons in music theory, history, and culture; and to a lesser extent, (d) student creativity through composition and arranging.

Remote or distance learning has existed as an option for music instruction since the late 19th century when professional artists offered lessons through the mail (Martin, 1999). The advent of various technologies provided further opportunities, including instruction on phonograph recordings in the early 1900s (Hash, 2016), over the radio by the 1930s (Sanders, 1990), and via television during the 1960s (Allen, 1966). Beginning in 1993, the University of Iowa utilized fiber-optic technology to deliver instrument master classes to public school students through a two-way audiovisual television system that allowed interaction between the instructor and students (Rees & Downs, 1995).

Today, P–12 students access online distance learning (ODL) in a variety of subjects including music via the World Wide Web. Parents can obtain applied instruction (e.g., Lessons in Your Home, 2020) and general music activities targeted to the age and ability level of their child (e.g., Music Together, 2020). P–12 teachers use the Internet to feature guest artists in their classrooms (e.g., Burrack, 2012; Hoffman & Carter, 2014) and to facilitate other types of learning. The Vermont Midi Project, now Music-COMP (Music Composition Mentoring Program), for example, began in 1995 to facilitate implementation of the National Standards for Music Education. Students develop original compositions through collaboration online with peers, teachers, and professional composers (Ball et al., 2004).

Remote instruction in P–12 education today might occur when schools must close due to weather or other conditions. For some students, remote learning might simply involve receiving a packet of instructions and materials from their school that they complete and return independently. Others will likely participate in some form of ODL, also referred to as eLearning by some educators (e.g., Illinois State Board of Education, 2019, 2020). Classes can be synchronous, meaning that they happen over a videoconferencing platform in real time, or asynchronous, which involves guided independent study around specific assignments and due dates (Sleator, 2010).

ODL presents both advantages and challenges. Advantages include facilitating instruction to remote areas, flexible scheduling, and reduced travel (Albert, 2015; Meyen et al., 1998; Welker & Berardino, 2005-2006). Some students also might feel that the online environment is a safer space compared to the traditional classroom (Huang, 2014). Challenges include ensuring access to technology for all students, especially in high-poverty (e.g., Warschauer et al., 2004) and rural (e.g., Sundeen & Sundeen, 2013) schools; maintaining privacy and security of online data and interactions, connectivity of networks, and firmware; and monitoring students’ Internet activity. In addition, teachers must comply with copyright laws (Meyen et al., 1998), maintain student motivation and engagement (Wexler, 2020), build pupils’ information literacy (Huang, 2014), and meet the needs of all learners in the online environment (e.g., Straub, & Vasquez, 2015). School administrators must support their work through ongoing professional development related to remote instruction (Castelo, 2020).

Several authors have cited these advantages and challenges when investigating ODL in music through synchronous (e.g., Dye, 2016) and asynchronous (e.g., Kenny, 2013) instrumental lessons and instruction in braille notation (Jacko et al., 2015), theory and composition (e.g., MacLeod, 2013; Schmidt-Jones, 2020), and teacher education (e.g., Albert, 2015). Convenience was one of the advantages of synchronous (Dammers, 2009) and asynchronous (Bayley & Waldron, 2020; Kenny, 2013; Waldron, 2013) eLearning cited most often, even for underserved students (Pike, 2017). In addition, ODL can offer flexibility and multimodal pedagogy through written materials, message board discussions, emails, and videos. The Internet facilitates these and other processes such as recording and file sharing, all of which might sustain students’ interest more than traditional instruction (Koutsoupidou, 2014).

Audio quality and delay is frequently a challenge of ODL in music, especially for synchronous applied lessons (e.g., Biasutti, 2018; Dammers, 2009; Koutsoupidou, 2014; Pike, 2017). Riley et al. (2016) examined the use of three videoconferencing platforms, Skype, PolyCom, and LOw LAtency (LOLA), as tools for synchronous ODL. Students experienced instruction in either a classical master class, jazz lesson, or old-time fiddle session. On a scale of 1 to 5, participants rated the overall effectiveness of LOLA (*M* = 4.25, *SD* = 0.77) higher than PolyCom (*M* = 3.06, *SD* = 1.15) and Skype (*M* = 2.80, *SD* = 1.25). Regardless, all three platforms exhibited issues with latency and clarity of sound.

Concerns surrounding audio quality might cause applied teachers to employ more verbal directions and less modeling compared to traditional face-to-face instruction (Dye, 2016). However, they might also plan lessons, experiment with different strategies, encourage student independence, and reflect more on their work to compensate for the inability to play simultaneously. Both instructors and students need to exhibit patience at the beginning. Pike (2017) found that it took novice teachers 8 weeks to adjust pacing and verbal instruction appropriately to the online medium.

Effective ODL in music can foster a high level of student motivation and on-task behavior (Dammers, 2009) and reduced the anxiety and insecurity some people feel in face-to-face lessons, especially in group settings (Bayley & Waldron, 2020). However, frustrations with technology may result in student attrition (Schmidt-Jones, 2020) and cause learners to feel isolated (Koutsoupidou, 2014) and unsure of progress (Bayley & Waldron, 2020). Some online music communities have found ways to overcome the latter by fostering social interaction through blogs, chat forums, and file sharing (Kenny, 2013; Waldron, 2013). Despite some teachers who felt that online lessons were equal to face-to-face instruction in terms of academic quality, several authors concluded that ODL in music was “currently inadequate” (Koutsoupidou, 2014, p. 253) and “could serve only [as] a supplement” (Dammers, 2009, p. 17) or “a nominally acceptable substitute” (Dye, 2016, p. 169).

## Need and Purpose

Although many opportunities for learning music exist online (e.g., Lessons in Your Home, 2020), most P–12 students continue to receive instruction through face-to-face interaction with teachers in applied studios or school music programs. Circumstances changed, however, in March 2020, when a novel Coronavirus designated as COVID-19 led to a global pandemic. By June 1, the number of infections worldwide exceeded 6,000,000 with over 370,000 deaths. Cases in the United States alone topped 1,700,000 with over 100,000 deaths reported by June 1 (World Health Organization, 2020). To curb the spread of the virus, U.S. schools in all 50 states and all U.S. territories moved to remote learning (RL) models in which students received instruction at home through ODL or other means for the remainder of the academic year (MCH Strategic Data, 2020).

With little or no preparation, P–12 teachers moved instruction from physical classrooms to remote online and offline platforms. Music educators followed suit and attempted to adjust goals and activities to fit RL while still meeting students’ needs. Although the situation was challenging for all teachers, music educators had to find ways of providing meaningful instruction in a subject that typically depends on students interacting throughout the learning process.

Unlike that discussed in the literature review, RL provided during the COVID-19 shutdown was essentially emergency teaching rather than the implementation of curricula planned, organized, and designed for distanced environments. Knowing how teachers approached and experienced these unprecedented circumstances will help identify best practices, suggest avenues for future research, and inform professional development around RL. This line of research will help music educators prepare for future emergency school closures and perhaps foster ways of incorporating RL into the regular curriculum. Therefore, the purpose of this study was to examine the practices, experiences, and perspectives of elementary and secondary school band directors in relation to RL during the COVID-19 shutdown of spring 2020. The following questions guided this research: (1) What technologies and materials did school band directors utilize in RL? (2) What learning activities and assessments did school band directors implement through RL? (3) What factors affected band students’ participation in RL? (4) What support and challenges did school band directors experience in relation to RL? (5) How did RL experiences vary among teachers in low-poverty versus high-poverty schools and at the elementary/middle school level versus high school level? and (6) What were the conditions and instructional practices of school band programs that experienced relatively high and consistent levels of student participation in RL?

## Method

### Survey

I developed a survey of RL in school bands after reviewing the literature and monitoring related topics discussed in Facebook groups, including Music Educators Creating Online Learning (42,100 members), E-Learning in Music Education (14,700 members), and Band Directors: A Professional Development Endeavor (28,400 members). Two music education faculty and one school band director reviewed the initial draft to validate organization and content. I made subsequent revisions based on their recommendations and piloted the survey for clarity and usability with student teachers (*N* = 18) who were involved in RL at the time as part of the COVID-19 shutdown.

The final draft of the survey consisted of 57 items divided into four sections. The first section contained items related to director and school demographics and utilized categories for school locales and poverty levels based on those of the National Center for Education Statistics (NCES). School locale designations included *city*: population 50,000+ inside a principal city; *suburban*: 50,000+ outside a principal city; *town*: 2,500 to 50,000; and *rural*: <2,500 (Provasnik et al., 2007). The NCES determines school poverty levels by the percentage of students eligible for free or reduced-price lunch (FRPL) under the National School Lunch Program. Classifications include *low* = 0%–25%, *mid low* = 25.1%–50%, *mid high* = 50.1%–75%, and *high* = 75.1%–100% (Hussar et al., 2020).

The second section of the survey identified materials, technologies, activities, and assessment practices utilized during the period of RL. The third section examined student participation, and the final section addressed challenges and professional development in relation to RL during the COVID-19 shutdown. The form included multiple-choice items, Likert-type scales, and three optional writing prompts at the end of Sections 2 through 4 that allowed participants to elaborate on their responses or share other thoughts on RL in school bands. The survey and response data are available in the Supplemental File in the online version of the article.

The survey took approximately 10 minutes to complete. Analysis involved descriptive and nonparametric statistics due to the nominal and ordinal nature of the data. In making comparisons between teachers at different grade levels (i.e., 4–8, 9–12), I sometimes eliminated responses from participants who taught a combination of elementary/middle school and high school grades so as not to confound differences between these levels. Minor discrepancies in *N*/*n* or percentage totals resulted from participants occasionally skipping items. The writing prompts allowed participants to elaborate on their responses and provide anecdotal accounts of their experiences. However, I did not analyze these data for this article.

### Survey Administration

I used Qualtrics software to administer the survey. The sample consisted of elementary and secondary school band teachers from the State of Illinois listed on a database compiled by a university band program. I used Excel to eliminate duplicate addresses of those who taught multiple grade levels to ensure that directors received only one invitation, each of which contained a unique link that participants could not share or reuse. The invitation went to 1,575 email addresses on May 4, 2020, about 6 weeks after the start of RL in the state’s P–12 schools. Several invitations (*n* = 203) bounced or failed to send, resulting in a sample of 1,372 instrumental music educators. The survey remained opened for 2 weeks with reminder emails sent to nonrespondents 7 days and 3 days before the deadline. The university institutional review board approved the procedures for this study.

## Results

### Demographics

The initial sample consisted of 474 participants for a response rate of 34.5%. Respondents included early career (1–5 years; *n* = 56, 11.8%), midcareer (6–15 years, *n* = 146, 30.8%), seasoned (16–25 years, *n* = 158, 33.3%), and late career (26+ years, *n* = 25, 5.3%) band directors. Their schools resided in cities (*n* = 47, 9.9%), suburbs (*n* = 187, 39.5%), towns (*n* = 174, 36.7%), and rural communities (*n* = 66, 13.9%) and included low (*n* = 163, 34.4%), mid low (*n* = 140, 29.5%), mid high (*n* = 97, 20.5%), and high (*n* = 71, 15.0%) poverty institutions. Chi-square analysis indicated no significant difference in the proportion of early career, midcareer, seasoned, and late career teachers in mid-low/low versus mid-high/high poverty schools, χ^2^(3, 470) = 3.34, *p* = .34, *V* = .05.

Some directors taught only elementary (grades 4–5, *n* = 16, 3.4%), middle school (grades 6–8, *n* = 103, 21.7%), or high school (grades 9–12, *n* = 122, 25.7%) bands, and others worked with a combination of these levels (*n* = 232, 48.9%). The number of students taught by each participant varied from 1 to 50 (*n* = 58, 12.2%), 51 to 100 (*n* = 116, 24.5%), 101 to 150 (*n* = 155, 32.7%), 151 to 200 (*n* = 76, 16.0%), and 200+ (*n* = 69, 14.6%).

### Instruction

Teachers provided remote instruction through eLearning (*n* = 372, 78.5%), nonelectronic delivery (e.g., physical materials circulated at distribution centers; *n* = 4, 0.8%), and a combination of these methods (*n* = 86, 18.1%). Respondents (*n* = 12, 2.5%) who indicated that they did not deliver remote instruction to band students concluded their participation in the survey at that point. Further analyses involved only teachers (*N* = 462) who provided remote instruction during the COVID-19 shutdown.

Remote instruction involved various platforms and software with videoconferencing, learning management systems, and noninteractive websites used by at least 70% of directors (see Table 1). Although the majority incorporated synchronous videoconferencing (*n* = 381, 82.5%), most held one (*n* = 210, 45.5%) or fewer (*n* = 98, 21.2%) meetings weekly. A smaller number held class 2 to 5 days per week (*n* = 73, 15.8%) or did not meet with students online (*n* = 81, 17.5%).

**Table 1.** Media Involved in Delivery of Remote Learning.

Platform	*n* ^a^	%
Video conferencing platform (e.g., Zoom, Google Hangouts)	373	80.7
Learning management system (e.g., Blackboard, Google Classroom)	362	78.4
Noninteractive websites (e.g., YouTube)	328	71.0
Music accompaniment software (e.g., SmartMusic, online play along tracks)	277	60.0
Physical materials (i.e., instruments, learning packets)	272	58.9
Interactive music learning web sites (e.g., MusicTheory.net)	215	46.5
Music notation software (e.g., Finale, Sibelius, MuseScore, NoteFlight)	186	40.3
Collaborative platform (e.g., Flipgrid, Screencast-O-Matic)	133	28.8
Audio editing software (e.g., Audacity, Garage Band)	104	22.5
Video editing software (e.g., Acapella for iPhone)	83	18.0
Assessment platform (e.g., Kahoot, Quizalize)	68	14.7
Telephone	54	11.7
Other	29	0.6

a *N* = 462. Participants could select multiple responses.

Ratings for frequency of whole-class, small-group, and individual sessions during RL ranged from 1 to 4 (*never, rarely, sometimes, often*). A Friedman analysis of variance indicated a significant difference in the frequency of whole-class, small-group, and individual student meetings among all directors, χ^2^(2, 456) = 10.997, *p* = .004. Post hoc analysis using Wilcoxon signed ranks tests with Bonferroni correction revealed a significantly higher frequency of small-group (*Z* = −3.12, *p* = .002, *r* = −.10) and individual (*Z* = −3.509, *p* < .001, *r* = −.12) sessions compared to whole-class meetings. In addition, a Mann-Whitney U test indicated that directors who taught only high school (grades 9–12, *n* = 119, 25.8%) held whole-class meetings significantly more frequently than teachers in only elementary and/or middle schools (grades 4–8, *n* = 248, 53.7%; *U* = 12,276.0, *p* = .007, *r* = −.14).

Directors rated six priorities of RL on a scale of 1 to 4 (*nonpriority, low, medium*, or *high priority*). Items ranked as high or medium priorities by most respondents included “maintaining students’ well-being” (*n* = 459, 99.4%), “maintaining motivation in music” (*n* = 440, 95.2%), “maintaining a sense of community” (*n* = 413, 89.2%), “developing individual musicianship” (*n* = 369, 79.9%), and “recruiting and retaining students” (*n* = 339, 73.4%). Almost all participants rated “preparing band repertoire” as a low priority or a nonpriority (*n* = 385, 83.3%) in this study.

The frequency of instructional activities varied as well. Those implemented often or sometimes by most respondents included practice assignments, music listening, and music theory/aural skills. However, more than 80% of bands rarely or never engaged in lessons related to composition or arranging, virtual ensembles, or sessions with guest artists or speakers (see Table 2). Many participants provided learners with choices of assignments (*n* = 286, 61.9%) and/or assessments (20.3%, *n* = 94), targeted instruction to various ability levels (*n* = 384, 83.1%), and modified or adapted instruction for special learners (*n* = 246, 53.2%).

**Table 2.** Frequency of Remote Learning Instructional Activities.

Activity	Never %	Rarely %	Sometimes %	Often %
Practice assignments	3.9	7.8	22.2	66.3
Music listening	6.0	11.9	45.5	36.2
Music theory/aural skills	19.2	26.3	32.1	21.3
Masterclass or lesson videos	31.5	22.0	30.4	15.3
Journals/personal reflection	36.2	20.7	23.5	19.0
Music history and culture	32.8	24.6	30.6	11.2
Composition/arranging	54.3	24.4	15.3	4.7
Virtual band/ensembles	69.2	14.4	11.0	4.1
Guest artists/speakers via video conferencing	76.6	12.5	7.3	3.4

*Note*. Arranged in descending order (sometimes + often).

### Assessment

Most directors assessed all (*n* = 324, 70.1%) or some (*n* = 74, 16.0%) students differently during RL compared to face-to-face instruction. Almost all participants (*n* = 405, 87.7%) utilized teacher assessments and instructed students to provide self (*n* = 235, 50.9%) and peer (*n* = 48, 10.4%) feedback less often. Some directors (*n* = 52, 11.3%) did not implement assessments during RL.

Assessment artifacts consisted of performance video or audio recordings (*n* = 403, 87.2%); reflections or essays not related to practicing (*n* = 237, 51.3%); screenshots or other evidence of completion (*n* = 224, 48.5%); practice logs, records, or journals (*n* = 173, 37.4%); worksheets (*n* = 136, 29.4%); and compositions/arrangements (*n* = 91, 19.7%). A handful of respondents (*n* = 18, 3.9%) did not collect assessment artifacts. Chi-square analysis found no significant difference in the proportions of artifacts utilized by participants who taught only high school (*n* = 119, 25.8%) or only elementary and/or middle school (*n* = 249, 53.9%), χ^2^(5, 979) = 8.14, *p* = .149, *V* = .04.

Evaluation (i.e., grading) of assignments consisted of complete/incomplete (*n* = 256, 55.4%), letter grades (*n* = 99, 21.4%), standards-based criterion (*n* = 37, 8.0%), and pass/fail (*n* = 27, 5.8%). Some teachers (*n* = 42, 9.1%) did not provide summative evaluation for assignments.

### Participation

Many directors (*n* = 336, 72.7%) stated that their institution provided some or all students with a device and/or Internet access (i.e., 1-to-1) on a regular basis (*n* = 291, 63.0%) or to facilitate RL during the COVID-19 shutdown (*n* = 145, 31.4%). A small number (*n* = 25, 5.4%) said that their school did not provide students with technology. Most teachers (*n* = 362, 78.4%) indicated that 76% to 100% of their students had Internet access outside of school. However, access varied depending on poverty level and location. Only 55.8% (*n* = 91) of teachers in mid-high/high poverty schools (*n* = 163) reported student access to the Internet at or above 76% compared to 90.2% (*n* = 276) in mid-low/low poverty schools (*n* = 296). In addition, 81.4% (*n* = 324) of teachers in nonrural schools (*n* = 398) indicated >76% student Internet access versus 63.6% (*n* = 41) at rural institutions (*n* = 66). Despite lower representation in the sample, directors from higher poverty and/or rural schools represented 86.1% (*n* = 87) of those reporting student Internet access at 75% or below (*n* = 101).

Respondents rated student participation as *total* (100%), *high* (80%–99%), *moderately high* (60%–79%), *moderate* (40%–59%), *moderately low* (20%–39%), or *low* (0%–19%). Data indicated participation as total (*n* = 10, 2.2%), high (*n* = 141, 30.5%), moderately high (*n* = 120, 26.0%), moderate (*n* = 71, 15.4%), moderately low (*n* = 100, 21.6%), and low (*n* = 21, 4.5%). A greater percentage of directors in mid-low/low poverty schools (*n* = 205, 69.3%) reported student participation at moderately high or above compared to those in mid-high/high poverty institutions (*n* = 61, 37.4%).

Administrators for 69.3% (*n* = 320) of respondents required students to participate in band during RL. However, high school administrators mandated participation significantly more often than those leading elementary and middle schools, χ^2^(1, 367) = 24.13, *p* < .001, φ = .26. Just 61.0% (*n* = 152) of directors teaching only grades 4 through 8 (*n* = 249) had required participation versus 86.6% (*n* = 103) working only with grades 9 through 12 (*n* = 119).

The sample (*N* = 462) described student participation on a scale of 1 to 4 ranging from *very sporadic* (*n* = 37, 8.0%) to *sporadic* (*n* = 115, 24.9%), *somewhat consistent* (*n* = 197, 42.6%), *consistent* (*n* = 98, 21.2%), or *very consistent* (*n* = 15, 3.2%). Respondents indicated significantly greater participation for lower (*n* = 296, 64.1%) versus higher (*n* = 163, 35.3%) poverty schools (*U* = 15,769.0, *p* < .001, *r* = −.30) and for programs requiring (*n* = 320, 69.3%) versus not requiring (*n* = 139, 30.1%) student participation (*U* = 19,644.5, *p* < .036, *r* = −.10). No significant difference was found for elementary/middle school only (*n* = 249, 53.9%) versus high school only (*n* = 119, 25.8%) directors (*U* = 13,807.5, *p* < .26, *r* = −.05).

I also examined conditions and instructional practices of respondents who indicated high/total and consistent/very consistent participation (*n* = 79, 17.1%). Almost all had at least 6 years of teaching experience (*n* = 77, 97.5%), worked in mid-low/low poverty schools (*n* = 73, 92.4%), had >76% of students with home Internet access (*n* = 77, 97.5%), and held videoconference sessions at least once per week (*n* = 57, 72.2%). In addition, almost all these respondents taught in a school that provided students with a device and/or Internet access (*n* = 77, 97.5%) and with administrators who required band students to participate in RL (*n* = 66, 83.5%).

Teachers who experienced high and consistent participation generally employed instructional activities and assessments at frequencies similar to those of other participants. Likewise, their priorities generally mirrored those of the total sample. However, these directors rated “developing individual musicianship” as a significantly higher priority than other respondents did (*U* = 10,623.0, *p* < .001, *r* = −.20).

### Teacher Experiences

Participants rated the amount of professional development received for RL from various sources on a scale of 1 to 4 (*none, a little, some, a lot*). Based on combined responses of some and a lot, other colleagues (*n* = 342, 74.0%) provided the most assistance, followed by school technology support (*n* = 246, 53.2%), school administration (*n* = 229, 49.6%), Facebook and/or similar platforms (*n* = 219, 47.4%), professional organizations (*n* = 210, 45.5%), and podcasts (*n* = 71, 15.4%).

The extent to which school band directors rated different challenges of RL ranged from *not at all* to *minor, moderate*, and *extreme* (see Table 3). Challenges rated as moderate or extreme by the majority of respondents included “sustaining remote learning to the end of the year” (*n* = 336, 72.7%) and “planning appropriate instruction feasible through remote learning” (*n* = 313, 67.7%). Likewise, >80% of participants identified “administrative support,” “teacher technology skills,” “copyright laws,” “Internet security,” and “teacher access to technology” as minor to nonexistent challenges during RL.

**Table 3.** Challenges of Remote Learning in Band.

Challenge	Not a Challenge %	Minor Challenge %	Moderate Challenge %	Extreme Challenge %
Sustaining remote learning to the end of the year	6.5	20.8	32.3	40.5
Planning appropriate instruction feasible through remote learning	8.9	22.9	40.3	27.5
Parental support	13.9	37.7	34.4	14.1
Modifying/differentiating instruction for special learners	18.6	33.1	31.8	16.5
Student access to instruments, music, and related supplies	23.8	40.5	25.5	10.2
Students’ technology skills	18.2	48.7	29.2	3.9
Student access to technology	24.9	47.8	20.6	6.7
Administrative support	55.2	24.9	13.4	6.3
Teacher technology skills	45.0	35.7	14.5	4.3
Copyright laws	62.6	23.8	9.1	4.1
Internet security	56.9	31.2	9.3	2.4
Teacher access to technology	74.7	17.7	6.3	1.1

*Note*. Arranged in descending order (moderate challenge + extreme challenge).

Teachers rated most challenges similarly regardless of grade or poverty level. However, directors in only grades 9 through 12 (*n* = 119, 25.8%) were challenged to a significantly higher degree with “student access to instruments, music, and related supplies” compared to those working only in grades 4 through 8 (*n* = 249, 53.9%; *U* = 12,166.5, *p* = .003, *r* = −.15). In addition, teachers in mid-high/high poverty schools (*n* = 163, 35.3%) faced significantly greater challenges with “student access to technology” (*U* = 14,655.0, *p* < .001, *r* = −.35), “parental support” (*U* = 18,229.5, *p* < .000, *r* = −.21), and “student access to instruments, music, and related supplies” (*U* = 19,499.0, *p* < .000, *r* = −.17) compared to their colleagues in mid-low/low (*n* = 296, 64.1%) poverty schools.

## Discussion

This study examined RL practices of school bands during the COVID-19 shutdown of spring 2020. Directors responded to items related to instruction and assessment, student participation, and personal challenges and perspectives. These data represent efforts made by music educators to continue teaching amid crisis and without the resources and preparation needed to develop curricula for RL platforms. Findings could have applications for future RL due to long-term school closures, emergency weather days, and the need to provide instruction during the summer or for students with extended absences. Readers should interpret results with caution and note the following limitations. First, the survey was not deeply rooted in extant literature due to a lack of studies on this topic. Second, findings could be specific only to teachers from the State of Illinois. Third, data indicating that only 2.5% (*n* = 12) of participants did not engage in RL might not reflect the population. Those who did not teach band during this period may have felt they had nothing to offer and chose not to complete the survey. Finally, small effect sizes for inferential statistics suggest that significant findings could be an artifact of sample size rather than practical differences.

### Instruction and Assessment

Most directors that held video conferencing sessions met with students once per week or less. Intermittent contact might have resulted from administrative policies that placed restrictions on videoconferencing due to security and privacy issues, focused on core classes, and lacked scheduled time for ensembles. In addition, the frequency with which teachers utilized private and small-group lessons versus whole-class meetings suggests that they found individualized instruction more effective for delivering feedback and monitoring student progress. Several participants also mentioned the inability of students to play together due to latency and poor sound quality from current technology. Although a handful of teachers attempted to use video editing software (e.g., Acapella) to create ensemble performances from individual recordings, most rarely or never engaged in this activity. Perhaps some directors found that creating virtual ensembles was too complicated or not beneficial to their students. Further development of technology is necessary before musicians will be able to play together in real time over the Internet (e.g., Riley et al., 2016).

Many respondents allowed students to choose assignments during RL. However, most of the options centered on performing, listening, and music theory. Students rarely or never composed or arranged music, responded to or reflected on music, or explored music history and culture. Perhaps some directors could take a broader approach during RL and include activities that involve creating and responding, as per standards by the National Association for Music Education (NAfME; 2014). Online platforms could facilitate collaborative composing or arranging among smaller groups (Biasutti, 2018; Cremata & Powell, 2015; Hopkins, 2015) and might engage students who do not have access to an instrument or who lack the independence or motivation to play alone.

Reasons for not composing or arranging during RL might relate to instructors’ lack of comfort or preparation in teaching these activities (Bell, 2003; Cremata & Powell, 2017; Menard, 2015) or the perception that creating music is not part of the ensemble curriculum. Other obstacles may include a shortage of instruction time and the need for notation software or other tools (Strand, 2006). Preservice instruction and inservice workshops on composing and arranging in face-to-face and virtual environments could help instrumental teachers strengthen their abilities in these areas (e.g., Hopkins, 2013).

Teachers should also reevaluate assessment practices to include more peer feedback and self-reflection. These strategies would provide an opportunity for students to respond to music and develop independence and would encourage metacognition (e.g., Pike, 2017). Peer assessment and collaborative composing/arranging could also help some students feel less isolated (Kenny, 2013; Koutsoupidou, 2014; Waldron, 2013) during RL and foster social interaction, which is a key element in the large-ensemble experience (Ebie, 2005).

### Participation

A few differences in teacher characteristics and instructional choices may have affected student participation. The finding that almost all directors with higher and more consistent participation had been teaching for at least 6 years might imply that those with experience were better able to adapt to the challenges of RL. They could also have had stronger relationships with students that helped them in fostering participation and shaping instruction. For example, these directors focused significantly more on individual student musicianship compared to the rest of the sample. Perhaps students found individualized performance activities more engaging and meaningful and felt a higher level of accountability compared to those who pursued other learning outcomes.

Regardless, variables outside teachers’ control (e.g., Sundeen & Sundeen, 2013; Warschauer et al., 2004) probably affected participation to the greatest extent. Data indicated that respondents with high and consistent participation worked mostly in mid-low/low poverty institutions and with students who almost always had access to a device and the Internet. In addition, most administrators in these schools required RL for students in band. Music educators will have to continue advocating for improved student access to technology and for policies that prioritize the arts as part of a “well rounded curriculum” (Every Student Succeeds Act, 2015, p. 172) regardless of delivery format.

Almost all respondents indicated that “maintaining students’ well-being” was a top priority during RL. This finding aligned with the Illinois State Board of Education’s (2020) mandate that schools “focus [on] keeping children emotionally and physically safe, fed, and engaged” and that they inflict “no educational harm” (p. 19). As a result, almost all schools followed further recommendations asking that “student grades [were] not lowered as a result of remote learning” (p. 19). This directive and many school administrators’ decisions to make band an optional class during this period might have resulted in students disengaging.

Other factors that could have hindered participation included students’ access to the Internet due to multiple people needing to use a single computer, student obligations in caring for younger siblings, and parents not allowing their child to play an instrument while other family members were working or schooling from home. Many teachers at higher poverty schools or in grades 9 through 12 experienced challenges with student access to instruments, music, and related supplies. Contributing circumstances probably included students sharing school-owned equipment and policies prohibiting them from returning to the building to retrieve instruments after the announcement to end in-person instruction.

Most directors found that sustaining RL to the end of the year was their greatest challenge. A lack of parental support, cited as a moderate to extreme challenge by 48.5% of respondents, likely exacerbated this problem in some cases. Continued problem solving, communication, advocacy, and policy development on the part of teachers, administrators, and music education organizations (e.g., National Federation of State High School Associations [NFHS] & NAfME, 2020) will be necessary to foster support and maintain instruction during times of RL.

### Equity

The finding that nearly 73% of directors worked in schools that provided some or all students with technology and/or Internet access might be surprising. One contributing factor could be that Illinois falls within the top 25% among states for per pupil spending on public education (World Population Review, 2020). It is also possible that teachers from schools that did not supply students with technology also did not implement RL and/or complete the survey. Regardless, results from this study support previous research (e.g., Sundeen & Sundeen, 2013; Warschauer et al., 2004) that found inequities in access to technology among students who attend high-poverty schools and/or live in rural communities. Directors in higher poverty schools also reported significantly greater challenges with parental support and student access to instruments and other materials than directors in lower poverty schools did. These conditions likely provide a partial explanation for why these teachers experienced lower and less consistent participation compared to their colleagues in more affluent areas. Continued work on the part of school systems and government agencies is necessary to provide all students with access to technology and other materials needed for learning online and offline. In the meantime, instrumental teachers will need to explore alternative ways to provide RL to underresourced students. Solutions could involve creating a series of learning packets that contain practice instructions, sheet music, worksheets, self-assessments, and other printed materials that students exchange in person or through the postal service.

Assuming reliable Internet access, directors in rural communities might find that RL could supplement work in the traditional classroom by providing lessons and master classes in the absence of other resources. Perhaps parent organizations could help fund these experiences, which would begin to equalize the disparity of musical opportunities available in metropolitan versus rural areas (Dammers, 2009; King et al., 2019).

### Future Considerations

Planning instruction for RL was the second highest rated challenge cited in this study. The success of RL in the future will depend on music educators developing plans, creating materials, building an infrastructure, and preparing students for online and offline instruction. Technology skills and access were not major challenges for directors in this study. In addition, they received at least some support for RL from colleagues, school administrators, and technical support personnel. Preservice preparation and inservice professional development should address topics related to RL, including (a) technology and other resources, (b) instruction and assessment, (c) fostering student participation, and (d) advocacy (e.g., NFHS, 2020). College courses and inservice workshops should also teach (e) strategies for improving equity among underserved and special needs learners in these environments. In this study, just over half of respondents modified or adapted instruction for special learners, suggesting that RL in band might not have met the needs of all students.

Most teachers rated “recruiting and retaining students for next year” as a medium or high priority in this study. To maintain band programs, directors will need to find ways of fostering social interaction, musical growth, and performance opportunities among students until COVID-19 is no longer a threat. As students return to school and in-person instruction, teachers might honor social distancing and other guidelines by the Centers for Disease Control and Prevention (2020) by focusing on musical fundamentals and instrument technique in small-group lessons. Ensemble rehearsals and performances could involve students playing outdoors (e.g., NFHS Music Committee & Sports Medicine Advisory Committee, 2020) and presenting chamber music (e.g., NFHS & NAfME, 2020) in formal and informal settings in the school and community. Resources and processes for safely fitting beginners to instruments will also be necessary. For example, KHS Musical Instruments (Jupiter) now offers a wind instrument try-out kit with plastic mouthpieces that teachers can clean and disinfect to prevent spreading germs among students (KMS Musical Instrument Company, 2020).

The COVID-19 shutdown of spring 2020 resulted in an unprecedented disruption in P–12 education that affected all disciplines, including instrumental music. However, this period also created opportunities for school band directors to incorporate (a) a wider range of technology; (b) more of a focus on individual musicianship; (c) lessons in music theory, history, and culture; and (d) student creativity through composition and arranging. Future research is needed to determine best practices for RL in instrumental music education. Quantitative and qualitative studies could examine (a) instructional strategies for teaching private or small-group lessons, (b) approaches to assessment including self-reflection and peer feedback, (c) perspectives of various stakeholders, and (d) methods for improving learning and participation, especially among underserved, underresourced, or special needs students. More work is also needed to develop materials and curricula for RL and technology to facilitate collaborative musical performance in real time. These efforts will help ensure continued progress during future shutdowns or when instrumental music students cannot attend the classroom in person.

## Supplemental Material

sj-pdf-1-jrm-10.1177_0022429420967008 – Supplemental material for Remote Learning in School Bands During the COVID-19 Shutdown Supplemental material, sj-pdf-1-jrm-10.1177_0022429420967008 for Remote Learning in School Bands During the COVID-19 Shutdown by Phillip M. Hash in Journal of Research in Music Education
