# Supplementary material for: Remote Learning in School Bands During the COVID-19 Shutdown
Source: J Res Music Educ. 2020 Dec 7;68(4):381–97. doi: 10.1177/0022429420967008 (PMC7723736; doi:10.1177/0022429420967008)
Supplement: sj-pdf-1-jrm-10.1177_0022429420967008 – Supplemental material for Remote Learning in School Bands During the COVID-19 Shutdown [file sj-pdf-1-jrm-10.1177_0022429420967008.pdf]

Online Supplemental File

Hash, P. M. (2021). Remote learning in school bands during the COVID-19 shutdown. *Journal of Research in Music Education*.

SURVEY FORM

Start of Block: Introduction

The purpose of this study is to examine school band directors' experiences, perspectives, and concerns with remote learning during the COVID-19 shutdown. Your participation in this study is anonymous and voluntary. You will not be penalized if you choose to skip parts of the study, not participate, or withdraw from the study at any time. The survey will take 8-10 minutes to complete. Submitting this survey indicates your consent to participate and that you are a citizen of the United States. You can print this form for your records. Please direct questions to Dr. Phillip Hash at pmhash@ilstu.edu. Questions related to your rights as a participant may be directed to Illinois State University Research Ethics & Compliance Office at (309) 438-5527 or IRB@ilstu.edu.

End of Block: Introduction

Start of Block: Demographic Information

How many years of teaching experience will you have at the end of the 2019-20 school year?

- ○ 1 - 5 years (11.8%)
- ○ 6 - 15 years (30.8%)
- ○ 16 - 25 years (33.3%)
- ○ 26+ years (5.3%)

In what type of school system do you teach?

- ○ Rural (Population less than 2,500) (13.9%)
- ○ Town (Population 2,500-50,000) (36.7%)
- ○ Suburban (Population 50,000+ outside a principal city) (39.5%)
- ○ City (Population 50,000+ inside a principal city) (9.9%)

Approximately what percent of students at your school receive free or reduced lunch (i.e., low income)?

- ○ 0% - 25% (low poverty school) (34.4%)
- ○ 25.1% - 50% (mid-low poverty school) (29.5%)
- ○ 50.1% - 75% (mid-high poverty school) (20.5%)
- ○ 75.1% - 100% (high poverty school) (15.0%)

What grade level(s) of band do you teach? (Click all that apply.)

- ☐ 4th - 5th grades (43.7%)
- ☐ 6th - 8th grades (67.9%)
- ☐ 9th - 12th grades (44.7%)

How many band students do you teach?

- ○ 1 - 50 students (12.2%)
- ○ 51 - 100 students (24.5%)
- ○ 101 - 150 students (32.7%)
- ○ 151 - 200 students (16.0%)
- ○ 200+ students (14.6%)

End of Block: Demographic Information

Start of Block: Instruction & Assessment

How did you deliver remote instruction to band students during the COVID-19 shutdown?

- ○ eLearning (i.e., online) (78.5%)
- ○ Non-electronically (i.e., physical learning packets) (0.8%)
- ○ Combination of eLearning and non-electronic instruction (18.1%)
- ○ I did not deliver remote instruction to band students (2.5%)

Skip To: End of Survey If How did you deliver remote instruction to band students during the COVID-19 shutdown? = I did not deliver remote instruction to band students

What platforms did you utilize to prepare and/or deliver remote learning in band? (Click all that apply.)

- ☐ Music notation software (e.g., Finale, Sibelius, MuseScore, NoteFlight) (40.3%)
- ☐ Music accompaniment software (e.g., SmartMusic, online play along tracks) (60.0%)
- ☐ Audio editing software (e.g., Audacity, Garage Band) (22.5%)
- ☐ Video editing software (e.g., Acapella for iPhone) (18.0%)
- ☐ Interactive music learning web sites (e.g., MusicTheory.net, Sight Reading Factory) (46.5%)
- ☐ Non-interactive web sites (e.g., YouTube) (71.0%)
- ☐ Video conferencing platform (e.g., Zoom, Skype, Google Hangouts) (80.7%)
- ☐ Collaborative platform (e.g., Flipgrid, Screencast-O-Matic) (28.8%)
- ☐ Learning management system (e.g., Blackboard, Google Classroom, Moodle, Schoology, Canvas) (78.4%)
- ☐ Assessment platform (e.g., Kahoot, Quizalize) (14.7%)
- ☐ Physical materials (i.e. instruments, learning packets) (58.9%)
- ☐ Telephone (11.7%)
- ☐ Other ____ (0.6%)

On average, how frequently were students invited/required to attend a session via video conferencing?

- Never (17.5%)
- Less than 1 day per week (21.2%)
- 1 day per week (45.5%)
- 2 - 4 days per week (12.6%)
- 5 days per week (3.2%)

To what degree was each of the following a priority during the COVID-19 shutdown?

	Not a Priority %	Low Priority %	Medium Priority %	High Priority %
Preparing band repertoire	51.3	32.0	12.6	3.7
Developing individual musicianship	3.5	16.0	41.8	38.1
Maintaining students' well-being	0.2	0.4	15.4	84.0
Maintaining a sense of community among students	0.9	9.3	31.2	58.2
Maintaining students' motivation in music	0.2	4.3	32.0	63.2
Recruiting & retaining Students for next year (2020-21)	7.1	19.0	37.9	35.5

How frequently did you incorporate each of the following into remote learning in band?

	Never %	Rarely %	Sometimes %	Often %
Whole class meetings	40.9	21.6	20.5	16.2
Small group meetings	31.9	21.1	30.2	15.7
Individual meetings	28.7	22.8	29.7	17.7
Practice assignments	3.9	7.8	22.2	66.3
Music theory/aural skills	19.2	26.3	32.1	21.3
Music history & culture	32.8	24.6	30.6	11.2
Composition/arranging	54.3	24.4	15.3	4.7
Music listening	6.0	11.9	45.5	36.2
Journals/personal reflection	36.2	20.7	23.5	19.0
Masterclass or lesson videos	31.5	22.0	30.4	15.3
Virtual band/ensembles	69.2	14.4	11.0	4.1
Guest artists/speakers via video conferencing	75.6	12.5	7.3	3.4

Which of the following did you incorporate into remote learning in band? (Click all that apply.)

- ☐ Student choice of assignments (61.9%)
- ☐ Student choice of assessments (20.3%)
- ☐ Instruction targeted to various ability levels (83.1%)
- ☐ Modifications/adaptions of instruction for special learners (53.2%)

Which types of assessment artifacts did you collect as part of remote learning in band? (Click all that apply.)

- ☐ Performance video or audio recordings (87.2%)
- ☐ Practice logs, records, or journals (37.4%)
- ☐ Student reflections or essays not related to practicing (51.3%)
- ☐ Compositions/arrangements (19.7%)
- ☐ Worksheets (29.4%)
- ☐ Screenshots or other evidence of completion (48.5%)
- ☐ I did not collect assessment artifacts (3.9%)

Which types of assessment did you provide/facilitate as part of remote learning in band? (Click all that apply)

- ☐ Teacher assessment (87.8%)
- ☐ Peer assessment (10.4%)
- ☐ Self assessment (50.9%)
- ☐ Students were not assessed (11.3%)

To what extent did you evaluate (i.e. grade) students differently in remote learning compared to the traditional classroom?

- ○ I evaluated all students differently in remote learning (70.1%)
- ○ I evaluated some students differently in remote learning (16.0%)
- ○ I evaluated students the same way in remote learning as in the traditional classroom (13.4%)

How were individual assignments/projects evaluated <u>during remote learning</u>?

- ◯ Letter grades (which might not have affected the final semester grade) (21.4%)
- ◯ Pass/Fail (5.8%)
- ◯ Complete/Incomplete (55.4%)
- ◯ Standards-based/-referenced evaluation (e.g. does not meet, approaching, meets, exceeds) (8.0%)
- ◯ No evaluation given (9.1%)

(Optional) Please elaborate on your answers above and/or share other thoughts on instruction and assessment through remote learning in band.

____

____

____

____

____

End of Block: Instruction & Assessment

Start of Block: Student Access & Participation

Approximately what percent of your band students have access to the internet outside of school?

- ◯ 0% - 25% (0.06%)
- ◯ 26% - 50% (3.5%)
- ◯ 51% - 75% (17.3%)
- ◯ 76% - 100% (78.4%)

What technology does your school provide students for use outside of school?

- My school provides some/all students with a device (i.e. 1-to-1) (63.0%)
- My school provided a device and/or internet access to some/all students for use during the COVID-19 shutdown (31.4%)
- My school does not provide students with a device and/or internet access (5.4%)

Did school administration require students to participate in remote learning in band?

- Yes (69.3%)
- No (30.1%)

Approximately what percent of your students participated in remote learning in band during the COVID-19 shutdown?

- 0% (none) (0.0%)
- 1% - 19% (low) (4.5%)
- 20% - 39% (moderately low) (21.6%)
- 40% - 59% (moderate) (15.4%)
- 60% - 79% (moderately high) (26.0%)
- 80% - 99% (high) (30.5%)
- 100% (all) (2.2%)

In general, how would you describe the consistency of band students' participation in remote learning during the COVID-19 shutdown?

- ○ Very Sporadic (8.0%)
- ○ Sporadic (24.9%)
- ○ Somewhat Consistent (42.6%)
- ○ Consistent (21.2%)
- ○ Very Consistent (3.2%)

(Optional) Please elaborate on your answers above and/or share other thoughts on band students' participation in remote learning during the COVID-19 shutdown.

____

____

____

____

____

End of Block: Student Access & Participation

Start of Block: Perspectives

To what extent were each of the following a challenge to remote learning in band.

	Not a challenge %	Minor challenge %	Moderate challenge %	Extreme challenge %
Student access to instruments, music, and related supplies	23.8	40.5	25.5	10.2
My access to technology	74.7	17.7	6.3	1.1
Student access to technology	24.9	47.8	20.6	6.7
My technology skills	45.0	35.7	14.5	4.3
Students' technology skills	18.2	48.7	29.2	3.9
Planning appropriate instruction feasible through remote learning	8.9	22.9	40.3	27.5
Modifying/differentiating instruction for special learners	18.6	33.1	31.8	16.5
Sustaining remote learning to the end of the year	6.5	20.8	32.3	40.5
Parental support	13.9	37.7	34.4	14.1
Administrative support	55.2	24.9	13.4	6.3
Copyright laws	62.6	23.8	9.1	4.1
Internet security	56.9	31.2	9.3	2.4

How much support/professional development related to remote learning did you receive from the following sources?

	None at all %	A little %	Some %	A lot %
School Administration	18.8	31.6	28.1	21.4
School Tech Support	17.7	28.8	28.6	24.7
Facebook and/or similar platforms	33.1	19.3	27.9	19.5
Podcasts	63.9	20.3	12.6	2.8
Professional organizations	19.5	34.6	32.7	12.8
Other colleagues	4.5	21.4	38.3	35.7

(Optional) What else would you like to share in relation to remote learning in band during the COVID-19 shutdown? Feel free to elaborate on any of your answers above and/or discuss topics not addressed in this survey.

____

____

____

____

____

End of Block: Perspectives
